# Evaluation of an Open Source Registration Package for Automatic Contour Propagation in Online Adaptive Intensity-Modulated Proton Therapy of Prostate Cancer

**DOI:** 10.3389/fonc.2019.01297

**Published:** 2019-11-27

**Authors:** Yuchuan Qiao, Thyrza Jagt, Mischa Hoogeman, Boudewijn P. F. Lelieveldt, Marius Staring

**Affiliations:** ^1^The Division of Image Processing, Department of Radiology, Leiden University Medical Center, Leiden, Netherlands; ^2^Department of Radiation Oncology, Erasmus MC Cancer Institute, Rotterdam, Netherlands; ^3^Intelligent Systems Department, Faculty of EEMCS, Delft University of Technology, Delft, Netherlands; ^4^Department of Radiotherapy, Leiden University Medical Center, Leiden, Netherlands

**Keywords:** intensity modulated proton therapy, image registration, open source software, elastix, prostate cancer

## Abstract

**Objective:** Our goal was to investigate the performance of an open source deformable image registration package, elastix, for fast and robust contour propagation in the context of online-adaptive intensity-modulated proton therapy (IMPT) for prostate cancer.

**Methods:** A planning and 7–10 repeat CT scans were available of 18 prostate cancer patients. Automatic contour propagation of repeat CT scans was performed using elastix and compared with manual delineations in terms of geometric accuracy and runtime. Dosimetric accuracy was quantified by generating IMPT plans using the propagated contours expanded with a 2 mm (prostate) and 3.5 mm margin (seminal vesicles and lymph nodes) and calculating dosimetric coverage based on the manual delineation. A coverage of *V*_95%_ ≥ 98% (at least 98% of the target volumes receive at least 95% of the prescribed dose) was considered clinically acceptable.

**Results:** Contour propagation runtime varied between 3 and 30 s for different registration settings. For the fastest setting, 83 in 93 (89.2%), 73 in 93 (78.5%), and 91 in 93 (97.9%) registrations yielded clinically acceptable dosimetric coverage of the prostate, seminal vesicles, and lymph nodes, respectively. For the prostate, seminal vesicles, and lymph nodes the Dice Similarity Coefficient (DSC) was 0.87 ± 0.05, 0.63 ± 0.18, and 0.89 ± 0.03 and the mean surface distance (MSD) was 1.4 ± 0.5 mm, 2.0 ± 1.2 mm, and 1.5 ± 0.4 mm, respectively.

**Conclusion:** With a dosimetric success rate of 78.5–97.9%, this software may facilitate online adaptive IMPT of prostate cancer using a fast, free and open implementation.

## 1. Introduction

Intensity-modulated proton therapy (IMPT) for prostate cancer treatment has the potential to deliver a highly localized dose distribution to the target volume. However, IMPT is also sensitive to treatment-related uncertainties that may distort the planned dose distribution. These include uncertainties in patient set-up, inter-fraction and intra-fraction variations in the shape and position of the target volume and organs at risk (OARs), and uncertainties in the range of the proton beams (1–5).

The uncertainties are usually accounted in the clinical-target-volume to planning-target-volume (CTV-to-PTV) margin, while proton-therapy specific effects need to be accounted for by including robustness in the optimization of the treatment plan (2–4). Both come at a price in terms of sparing of OARs. Therefore, ideally, these uncertainties should be tackled at each treatment fraction by re-optimizing the treatment plan, based on a new CT scan-of-the-day. This requires new contours for the target and OARs. Manual re-contouring, however, takes a substantial amount of time, which would give rise to new shape and position uncertainties of the target and OARs. Fast automatic methods are therefore mandated.

Deformable Image Registration (DIR) provides an efficient way to automatically re-contour the repeat CT scan by establishing the spatial correspondence with the planning CT scan. The manual contours from the planning CT scan are then propagated to the repeat CT scan, thereby compensating for anatomical changes that may have occurred between the time of the acquisition of the planning CT scan and the time of delivery. In combination with fast IMPT treatment replanning this enables the reduction of margins and robust planning parameters. The important step of DIR in an online-adaptive IMPT procedure (re-contouring, re-planning, patient-specific QA), however, is currently rather time-consuming (6, 7) and DIR at the treatment delivery unit is time-critical and has high time-efficiency demands. In this paper we therefore developed and evaluated a fast and automatic DIR method, and performed a dosimetric evaluation for IMPT.

Many DIR algorithms implemented in commercial or open source software packages could be used clinically (8, 9). Commercial software packages are, however, frequently black boxes for users and have limited choices for parameter customization (6, 9). Open source packages are much more flexible and provide fully customizable algorithms (10–12). Moreover, they support the fundamental scientific principle of reproducibility, sharing of knowledge and thereby promote opportunities for scientific advancement (13–15).

Currently, most research focused on the validation of DIR for radiation therapy in terms of dose accumulation for the prostate (16–18) or other anatomical areas (19, 20), as well as in terms of the geometric accuracy (9, 21). Dosimetric or geometric validations were performed mostly independently or sometimes jointly, while the time cost of DIR was ignored. However, the time cost of image registration is also important for online adaptive IMPT (7, 10, 11). Kupelian et al. (22) found the prostate having a shift exceeding 5 mm in 15% of the fractions when duration exceeded 30 s and for some cases the motion was even larger than 10 mm when duration exceeded 240 s. Van der Wielen et al. (23) reported that the inter-fractional prostate motions not only have translational, but also rotational components and deformations. Moreover, if lymph nodes are included in the target volume then the differential motion of the prostate and lymph nodes have to be taken into account as well (24). Therefore, these deformations should be accounted for by DIR within a reasonable time span, especially when using a small margin like 3 mm (25). Conventional registration methods took from around 10 min to 1 h (26–29). Although recent advances in deep learning based image registration yield promise for real-time algorithms (30–32), this has not yet been shown for prostate CT radiotherapy. To our knowledge, the validation of open source packages on registration accuracy has not been investigated so far for prostate cancer in IMPT, in terms of the combination of geometric accuracy, dosimetric evaluation and runtime. The presented package, our software elastix, is open source and freely available for clinical application, research and further development. We compared its performance to another open source registration package: ANTs (https://github.com/ANTsX/ANTs) (33).

## 2. Materials and Methods

### 2.1. Patients and Imaging

Eighteen patients treated for prostate cancer with intensity-modulated radiation therapy were included in this study (1, 3). All patients gave their consent before being enrolled in a phase II dose-escalation trial delivered with moderately hypo-fractionated pelvic IMRT at Haukeland University Hospital, Bergen, Norway. The trial had been approved by the local ethical committee before enrollment starting in 2007. The ethics committee is REC West, the western Norway regional committee for medical and health research ethics (number 2006-15727). A planning CT and 7–10 repeat CTs evenly distributed throughout their treatment course were acquired out-of-room for each patient using a Philips Brilliance Big Bore CT scanner and anonymized with DicomWorks (Software Version: 2.2.1). The prostate, seminal vesicles, lymph nodes, and main OARs (rectum and bladder) of all patients were within the field-of-view. Geometric evaluation of DIR was performed using all images, while dosimetric evaluation was performed on a subset of 11 patients for which manual delineations of the bowels and femoral heads were available (needed for treatment planning). Each CT scan contained 90–180 slices and were reconstructed with a slice thickness of 2-3 mm. Each slice was of size 512 ×512 pixels and had an in-slice pixel resolution in the range from 0.84 ×0.84 mm to 0.95 ×0.95 mm. Golden fiducial markers (2 to 3) were implanted in the prostate for daily set-up to ensure an accurate alignment of the target with the treatment beams (34).

For each CT scan, the prostate, seminal vesicles, lymph nodes, bladder and rectum were delineated by an expert, and independently reviewed by another expert (1). The original images and delineations are in DICOM-RT format and were converted to meta image format and VTK meshes using MevisLab (http://www.mevislab.de/).

### 2.2. Image Registration

In this study, deformable image registration was performed using the open source software package elastix (12). This software is freely available from http://elastix.isi.uu.nl under the liberal Apache 2.0 license. All experiments were performed on a workstation with 64 GB memory, Linux system and an Intel Genuine i7-6850K CPU with 12 cores running at 3.6 GHz, utilizing only the CPU, without GPU acceleration.

For each patient, the planning CT scan (moving image) was registered to the repeat CT scans (fixed image), and then the manual delineations were propagated from the planning CT scan based on the corresponding deformations generated by DIR. The detailed procedure is as follows:

1. Preprocessing: A mask of the torso was generated automatically using in-house software Pulmo (commercialized by Medis specials, Leiden, The Netherlands), to eliminate the influence of the couch on image registration quality (35), and it was used for cropping the images. Each original image was cropped around the torso mask, using a 2 voxel margin, to further accelerate DIR. This preprocessing took much less than 1 s and is excluded in the runtime measures reported below.
2. Image registration: Image registrations were initialized based on the centers of gravity of the bony anatomy (tissue with HU > 200) of the cropped fixed and moving image. Then an affine registration was applied to tackle large movements of the organs, followed by DIR to compensate for local deformations. Image registration is formulated as an optimization problem:
  (1) $$\begin{array}{l} \hat{\mu }=\arg\underset{\mu }{\min}C\left(I_{F},I_{M}{}^{\circ}\;T\left(x,\mu \right)\right), \end{array}$$
  where *I*_*F*_ and *I*_*M*_ are the fixed and moving image, respectively, ***x*** is an image voxel location and ***T***(***x***, ***μ***) is a coordinate transformation parameterized by ***μ***. In this paper, an accelerated version of adaptive stochastic gradient descent (36) was used for iterative optimization of Equation (1). The number of iterations was varied between 100, 500, 1,000, and 2,000 iterations per resolution in the experiments below, in order to inspect the relation between DIR quality and runtime. A B-spline transformation model (37) was chosen and a fast recursive implementation was used (38). With the recursive B-spline, the original B-spline interpolation at a spatial coordinate ***x***∈*R*^*D*^,
  (2) $$\begin{array}{l} u\left(x\right)=\underset{\text{k}\in Z^{n}\left(x\right)}{\sum }B^{n}\left(x-\text{k}\right)\mu \left(\text{k}\right), \end{array}$$
  can be rewritten as:
  (3) $$\begin{array}{r} u\left(x\right)=\underset{{\text{k}}_{1}\in Z^{n}\left(x_{1}\right)}{\sum }\beta^{n}\left(x_{1}-{\text{k}}_{1}\right)\underset{{\text{k}}_{2}\in Z^{n}\left(x_{2}\right)}{\sum }\beta^{n}\left(x_{2}-{\text{k}}_{2}\right)\\ \underset{{\text{k}}_{D}\in Z^{n}\left(x\right)}{\sum }\beta^{n}\left(x_{D}-{\text{k}}_{D}\right)\mu \left(\text{k}\right), \end{array}$$
  where ***B***^*n*^(·) is the multi-dimensional B-spline polynomial of order *n*, *Z*^*n*^(·) is its support region, ***k*** is the multi-dimensional index, ***μ***(***k***) are the control point coefficients, and *D* is the image dimension. Here, we took advantage of the separability property of the B-spline leading to less computations. Equation (3) can be computed in a recursive manner, see the paper (38). Template Meta Programming, which is a programming paradigm to enable the compiler to generate efficient assembly code, was used to implement the recursive formulation of the B-spline transformation. This resulted in approximately 2 times faster registration performance than the default implementation (38). We used mutual information as a similarity measure (39). Multi-threading and parallellization of parts of the software in combination with further software optimization were applied to the software for further acceleration. A three level multi-resolution scheme was chosen to deal with local minima and to reduce calculation burden: the images were smoothed using a Gaussian filter with standard deviations of 2, 1, and 0.5 mm. Detailed parameter settings are available at the elastix parameter file database found at the elastix website.
3. Automatic contour propagation: After image registration, the deformation field generated from the previous step was applied to the manual delineations of the planning scan to generate a new contour of the scan of the day. This step took substantially less than 1 s and was also excluded from the runtime measures reported below.

Standard practice in image-guided radiotherapy is to use implanted intra-prostatic markers for daily patient alignment. Therefore, the proposed DIR approach will be compared to this default strategy, i.e., marker based translation, MBT in short.

Registration with this procedure was compared with another state-of-the-art open source registration tool: ANTs. As ANTs is widely used in multimodality neuroimaging registration (40) and lung CT image registration (41), it is also applied to head and neck proton therapy (42), radiation therapy on primary tumor vasculature (43) and prostate cancer radiation therapy (44). We compared the proposed method to the latest release of ANTs (v2.3.1) on the same machine with the same preprocessing scheme as detailed above. Mutual information is chosen as a dissimilarity measure with 32 as the number of bins for computing mutual information. Random sampling is applied, using 25% of all voxels to compute the dissimilarity measure. The deformation field is modeled with the diffeomorphic BSplineSyN model with a grid size of 0.3. For 3 resolutions, the number of iterations is set to 80, 80, and 60, respectively. The smoothing and shrinking factors are set to 3 ×2 ×1 and 4 ×2 ×1, respectively. These parameters are derived from Cao et al. (44) and manually tuned a lot for prostate CT image registration.

### 2.3. Evaluation Measurements

For quantitative evaluation of the automatic DIR method we considered several aspects, such as runtime, recontouring quality and dosimetric coverage. Runtime is measured by the system clock, in seconds. The recontouring quality of the prostate, seminal vesicles, lymph nodes, bladder and rectum is measured by comparing the automatically propagated contour from the planning CT scan with the manual delineation of the repeat CT scan. As a first measure we consider the Dice Similarity Coefficient (DSC) (45):

(4) $$\begin{array}{l} \text{DSC}=\frac{2|R_{M}\cap R_{F}|}{\left|R_{M}|+|R_{F}\right|}, \end{array}$$

where ****R*_*F*_*** and ****R*_*M*_*** are the manually delineated regions in the repeat CT scan image and the propagated region in the planning CT scan image, respectively.

Two types of symmetric surface distances are used, namely the mean surface distance (MSD) and the 95% percentile Hausdorff distance (95%HD). Let ***F*** = {*a*_1_, *a*_2_, …, *a*_*n*_}, and ***M*** = {*b*_1_, *b*_2_, …, *b*_*m*_} represent the mesh points from two surfaces, their definitions are as follows (46):

(5) $$\begin{array}{l} \text{MSD}=\frac{1}{2}\left(\frac{1}{n}\overset{n}{\underset{i=1}{\sum }}d\left(a_{i},M\right)+\frac{1}{m}\overset{m}{\underset{i=1}{\sum }}d\left(b_{i},F\right)\right), \end{array}$$

(6) $$\begin{array}{l} 95\%\text{HD}=\max\left\{\text{PERC}95\left(d\left(a_{i},M\right)\right),\text{PERC}95\left(d\left(b_{i},F\right)\right)\right\}, \end{array}$$

in which $d\left(a_{i},M\right)=\underset{j}{\min}∥b_{j}-a_{i}∥$. Both distances are computed in 3D. The geometrical success rate γ is defined as the percentage of registrations which has a MSD < 2mm (slice thickness) for the prostate: γ = *n*{MSD < 2mm}/*N*, where *N* = 159 is the total number of registrations.

To measure the dosimetric impact of differences in manual delineations and automatically re-contoured delineations, IMPT plans were generated on each repeat CT scan for both sets of delineations (manual and automatic) for the 11 patients where delineations of the femoral heads and bowels are available. To evaluate the difference between the two delineations and the effect these differences have on the dose distributions, both IMPT plans are evaluated on the manual contours, which therefore acts as the ground truth. All IMPT plans were generated using Erasmus-iCycle, an in-house developed treatment planning system (47, 48). Erasmus-iCycle uses a multi-criteria optimization to generate a clinically desirable Pareto optimal treatment plan on the basis of a wishlist consisting of hard constraints and objectives. A small margin is used (2 mm around the prostate and 3.5 mm around the seminal vesicles and lymph nodes) to compensate for inevitable inaccuracies of the contour-propagation and to account for intra-observer variations in the manual contouring. Note that without the envisioned online-adaptive approach the margins are far from sufficient to account for shape and positions changes of the target volume, for which clinically a margin of 7 mm is used (3, 49, 50). Dose was prescribed according to a simultaneously integrated boost scheme in which the high-dose target volume (prostate + 2 mm margin) was assigned 74 Gy and the low-dose target volume (lymph nodes and seminal vesicles + 3.5 mm margin) 55 Gy, to be delivered using two laterally opposed beams. The optimization ensures that at least 98% of the target volumes receive at least 95% of the prescribed dose (*V*_95%_ ≥ 98%). To avoid overdose the optimization ensures that less than 2% of the target volumes receive more than 107% of the highest prescribed dose (*V*_107%_ ≤ 2%). Target dose conformity and low dose to the OARs was achieved by including artificial rings around the target and including OARs in the wishlist (51). For the recontouring to be clinically acceptable the automatically generated treatment plans should still fulfill these criteria. Plans from automatic recontouring are therefore evaluated based on the manual contours. The clinical success rate η is defined as the percentage of registrations for which the prostate directly meets the dose treatment criteria: η = *n*{*V*_95%_ ≥ 98%}/*N, N* = 93. A second more conservative measure of clinical success is when all target volumes (the prostate, seminal vesicles and lymph nodes) meet this dosimetric criterium: CSR = *n*{(_*V*_95%_ ≥ 98%)Prostate_ AND (_*V*_95%_ ≥ 98%)SV_ AND (_*V*_95%_ ≥ 98%)LN_}/*N, N* = 93, where SV is seminal vesicles and LN is lymph nodes, respectively.

## 3. Results

### 3.1. Image Registration Performance

**Figure 1:**
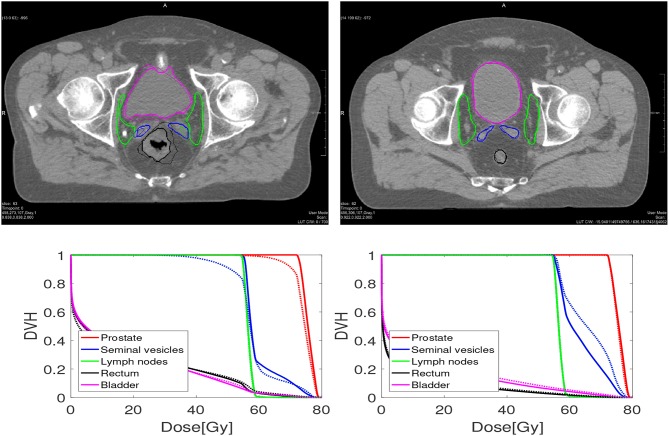
An example of one case **(Left)** where the dosimetric criteria was met for elastix, and one case **(Right)** where not. The bottom figures are dose volume histograms. The solid line represents the manual contouring results while the dotted line is the automatically propagated one with the setting of 100 iterations. For the prostate (left and right), the MSD is 2.26 and 1.12 mm, while *V*_95%_ is 90.8% and 99.8%, respectively. For the seminal vesicles **(Left, Right)**, the MSD is 2.74 and 1.00 mm, while *V*_95%_ is 88% and 100%, respectively. For the lymph nodes **(Left, Right)**, the MSD is 1.45 and 0.99 mm, respectively, while *V*_95%_ both are 100%.

Examples of automatically propagated contours using DIR are given in Figure 1. Table 1 presents the overlap after DIR for different numbers of iterations. For the prostate, we obtained a DSC of 0.88 ± 0.03 for each patient and all settings, and a similar overlap for the lymph nodes. The most difficult structures are the seminal vesicles, which have small volume and only achieved an overlap of 0.66 ± 0.16 for 100 iterations, and 0.67 ± 0.13 when the number of iteration is at least 500. For the OARs, we obtained a DSC of 0.77 ± 0.07 for the rectum and 0.88 ± 0.09 for the bladder for 100 iterations, and small improvements are observed when the number of iterations increased to at least 500. DSC scores generally improved from 100 to 500 iterations, but not after that. Compared to MBT we observe a 20% increase in DSC of seminal vesicles when using the proposed DIR method.

**Table 1:** Dice overlap of different organs for different elastix registration settings.

	**Prostate**	**Seminal vesicles**	**Lymph nodes**	**Rectum**	**Bladder**
**Nr. it**.	**Mean ± std**	**Mean ± std**	**Mean ± std**	**Mean ± std**	**Mean ± std**
MBT	0.88 ± 0.03	0.55 ± 0.21	0.86 ± 0.05	0.71 ± 0.08	0.78 ± 0.09
Affine	0.85 ± 0.08	0.46 ± 0.25	0.90 ± 0.04	0.71 ± 0.08	0.78 ± 0.09
100	0.87 ± 0.05	0.63 ± 0.18	0.89 ± 0.03	0.76 ± 0.06	0.86 ± 0.09
500	0.87 ± 0.05	0.64 ± 0.17	0.88 ± 0.03	0.77 ± 0.06	0.87 ± 0.09
1,000	0.87 ± 0.05	0.64 ± 0.17	0.88 ± 0.03	0.77 ± 0.06	0.87 ± 0.09
2,000	0.87 ± 0.05	0.65 ± 0.16	0.88 ± 0.03	0.78 ± 0.06	0.88 ± 0.10

**Figure 2:**
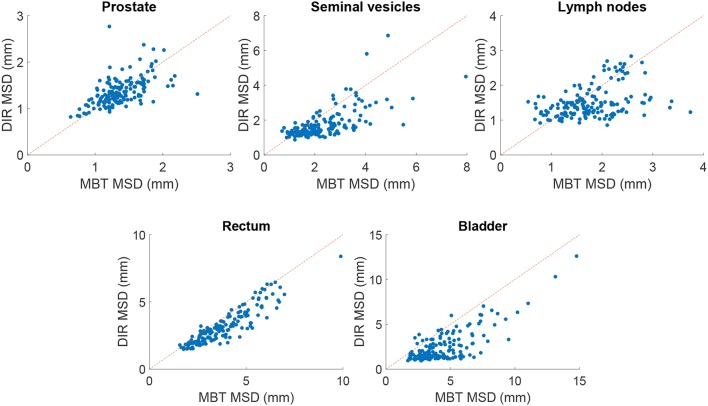
Scatter plot of the MSD in mm of elastix DIR with 100 iterations compared with the results of MBT. The red line indicates the line of no change, i.e., points below this line indicate improvement of DIR over MBT.

The mean surface distance (MSD) between the propagated and manually delineated contours are shown in Table 2. Note that for an increasing number of iterations the MSD slightly increased for the prostate and lymph nodes, likely due to the reduction in MSD of the bladder, rectum and seminal vesicles. However, also note that the MSD of the target organs were smaller than 1.8 mm which was within one voxel (0.9 ×0.9 ×2 mm). The 95% Hausdorff distance between the propagated and manual contour is shown in Table 3, which shows a similar pattern as the MSD. The geometrical success rate of the registrations was 96% (153 in 159) for DIR using 100 iterations and 96% (152 in 159) for DIR using more iterations, while this value was 77% (122 in 159) for affine registration. From Figure 2, we can see that DIR improved substantially compared to MBT, especially for the seminal vesicles, rectum and bladder.

**Table 2:** Mean surface distance (mm) of different organs for different elastix registration settings.

	**Prostate**	**Seminal vesicles**	**Lymph nodes**	**Rectum**	**Bladder**
**Nr. it**.	**Mean ± std**	**Mean ± std**	**Mean ± std**	**Mean ± std**	**Mean ± std**
MBT	1.36 ± 0.31	2.29 ± 1.10	1.76 ± 0.59	3.82 ± 1.41	4.40 ± 2.13
Affine	1.68 ± 0.85	2.98 ± 1.82	1.30 ± 0.48	3.95 ± 1.46	4.45 ± 2.11
100	1.42 ± 0.48	1.97 ± 1.22	1.46 ± 0.44	3.29 ± 1.31	2.92 ± 1.90
500	1.42 ± 0.48	1.90 ± 1.19	1.50 ± 0.44	3.20 ± 1.27	2.61 ± 1.81
1,000	1.42 ± 0.46	1.88 ± 1.16	1.53 ± 0.43	3.15 ± 1.27	2.47 ± 1.73
2,000	1.43 ± 0.49	1.87 ± 1.16	1.55 ± 0.43	3.10 ± 1.27	2.35 ± 1.67

**Table 3:** 95% percentile Hausdorff distance (mm) of different organs for different elastix registration settings.

	**Prostate**	**Seminal vesicles**	**Lymph nodes**	**Rectum**	**Bladder**
**Nr. it**.	**Mean ± std**	**Mean ± std**	**Mean ± std**	**Mean ± std**	**Mean ± std**
MBT	3.17 ± 0.90	5.47 ± 2.65	4.02 ± 1.54	11.47 ± 5.47	12.54 ± 7.06
Affine	3.97 ± 1.83	6.57 ± 3.46	3.19 ± 1.19	11.89 ± 5.66	12.41 ± 6.62
100	3.35 ± 1.19	4.76 ± 2.77	3.57 ± 0.99	10.83 ± 5.93	8.91 ± 6.76
500	3.46 ± 1.43	4.65 ± 2.73	3.72 ± 0.97	10.69 ± 5.99	7.95 ± 6.54
1,000	3.49 ± 1.53	4.64 ± 2.75	3.81 ± 0.96	10.62 ± 6.08	7.56 ± 6.39
2,000	3.54 ± 1.77	4.65 ± 2.82	3.88 ± 0.96	10.58 ± 6.17	7.24 ± 6.23

**Figure 3:**
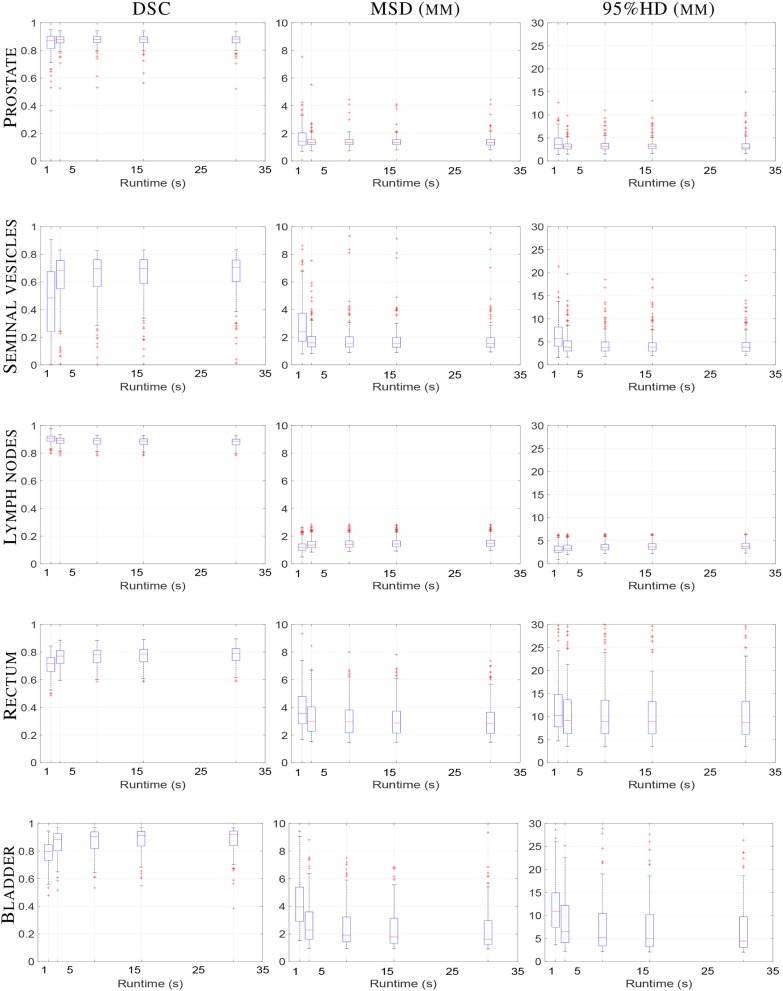
Boxplot of elastix registration performance against runtime in seconds. From left column to right column the DSC (unit-less), MSD (mm) and 95%HD (mm) are shown, respectively. From top to bottom the prostate, seminal vesicles, lymph nodes, rectum and bladder are shown, respectively. Within one boxplot, from left to right the affine registration and B-spline registrations with 100, 500, 1,000, and 2,000 iterations are shown, respectively. Each boxplot contains results of 159 registrations.

The total runtime in seconds for each registration setting was 3.1 ± 0.2, 8.8 ± 0.2, 16.1 ± 0.4 and 30.5 ± 0.4 s, for 100, 500, 1,000, and 2,000 iterations, respectively. Figure 3 illustrates the registration accuracy with respect to the mean runtime for different anatomical structures. Boxplots of the Dice overlap, mean surface distance and 95% Hausdorff distance are shown for all registrations (*N* = 159).

**Figure 4:**
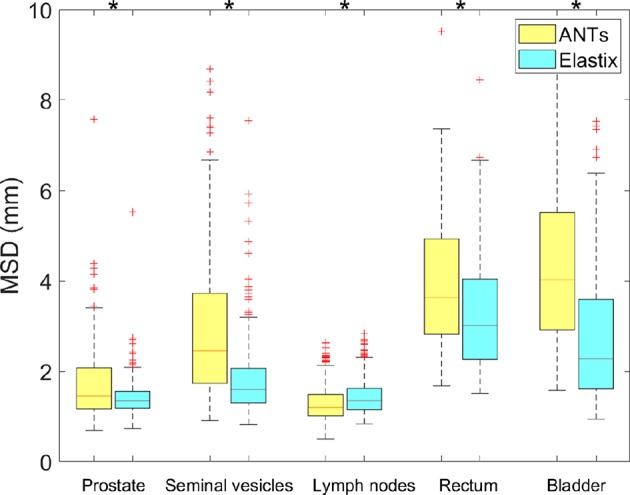
Boxplot of MSD from elastix DIR 100 iterations compared to ANTs for different organs. Star above boxes means a statistical difference of a Wilcoxon signed-rank test between the two methods.

The geometric results of ANTs are presented in Table 4 and Figure 4. The results shown in Table 4 present the geometric measures for each organ. It shows elastix outperformed ANTs for all different measures at different anatomical structures except for a 0.01 DSC difference for the lymph nodes. The runtime is about 10^3^ times faster for elastix compared to ANTs (3,659 ± 2,747 s). Runtime of ANTs is majorly influenced by the parameter settings, especially by the grid size. Larger grid size needs less runtime for ANTs, however, the accuracy will be sacrificed. The boxplots of the difference of each measure between elastix and ANTs are given in Figure 4. All results show statistical difference when using a Wilcoxon signed-rank test at *p* = 0.05.

**Table 4:** Geometric results compared to ANTs.

**Organs**	**Tools**	**DSC**	**MSD (mm)**	**95%HD (mm)**
	ANTs	0.85 ± 0.08	1.72 ± 0.87	4.12 ± 1.92
Prostate	elastix	**0.87 ± 0.05**	**1.42 ± 0.48**	**3.35 ± 1.19**
	ANTs	0.48 ± 0.25	3.07 ± 1.87	6.76 ± 3.53
Seminal vesicles	elastix	**0.63 ± 0.18**	**1.97 ± 1.22**	**4.76 ± 2.77**
	ANTs	0.90 ± 0.04	**1.33 ± 0.48**	**3.24 ± 1.20**
Lymph nodes	elastix	0.89 ± 0.03	1.46 ± 0.44	3.57 ± 0.99
	ANTs	0.71 ± 0.08	4.01 ± 1.47	11.99 ± 5.61
Rectum	elastix	**0.76 ± 0.06**	**3.29 ± 1.31**	**10.83 ± 5.93**
	ANTs	0.78 ± 0.09	4.56 ± 2.14	12.63 ± 6.65
Bladder	elastix	**0.86 ± 0.09**	**2.92 ± 1.90**	**8.91 ± 6.76**

*For elastix, the results of 100 iteration for B-spline registration are reported. The bold value means the best results achieved by each method*.

### 3.2. Dosimetric Validation

All treatment plans from elastix were evaluated by visual inspection of the dose distributions, the dose-volume histograms (DVHs) of the target volumes and OARs, and the clinical constraints. For the prostate, seminal vesicles and lymph nodes, we report the *V*_95%_ and *V*_107%_ of each treatment plan that used the DIR-generated contours. For the rectum, we consider *V*_45*Gy*_, *V*_60*Gy*_, *V*_75*Gy*_, and *D*_*mean*_, while for the bladder *V*_45*Gy*_, *V*_65*Gy*_ and *D*_*mean*_ are used, where *D*_*mean*_ means the average dose to the structure.

**Figure 5:**
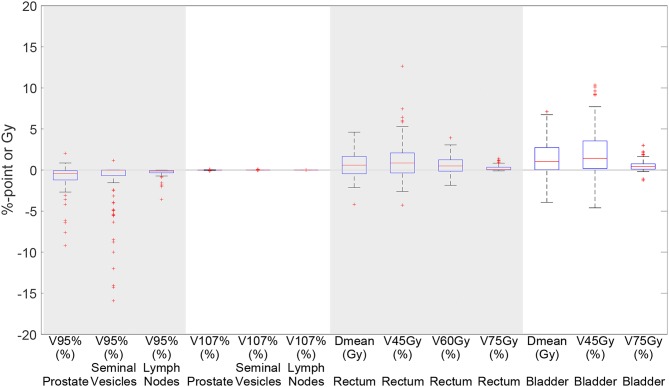
Boxplot depicting the difference in dosimetric parameters between the automatically generated delineations and the manual delineations in the treatment plan based on the automatically generated delineations using elastix 100 iterations for 93 scans. Each box plot indicates the median and the 25th and 75th percentiles of the obtained differences. The line depicts the remaining differences which are not outliers. Values are defined outliers if they are more than 1.5 times the distance between the 25th and 75th quartiles away from the quartiles. The red marks indicate the outliers.

Figure 5 shows a boxplot depicting the difference in dosimetric parameters between the automatically generated delineations (100 iterations setting) and the manual delineations, in the treatment plan that was based on the automatically generated delineations. We can see that for all dosimetric parameters the median of the differences are close to 0. However, there are some scans for which larger differences occur for especially the *V*_95%_ of the seminal vesicles.

**Figure 6:**
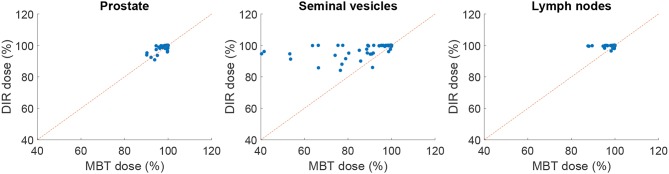
Scatter plot comparing elastix DIR (100 iterations) with MBT, in terms of dosimetric coverage (*V*_95%_). The red line indicates the line of no change, i.e., points above this line indicate improvement of DIR over MBT.

Table 5 shows the percentage of scans for which *V*_95%_ ≥ 98% and *V*_107%_ ≤ 2% for the treatment plans based on the automatically contoured structures. Note that the success rate when using the manual delineations is close to 100% for all organs. As one can see, DIR using 100 iterations obtained a success rate of 89.2% for the prostate and 78.5% for the seminal vesicles, and these numbers are improved to 89.2% and 88.2%, respectively for 500 iterations. The conservative success rate based on all three target volumes increased from 68.8% to 77.4%, for 100 and 500 iterations, respectively. For MBT, the CSR was only 50%, meaning that in 50% of the cases manual interaction during quality control is warranted. Compared to MBT, DIR improved the success rates with 9.4%, 26.6%, and 8.5% for the prostate, seminal vesicles, and lymph nodes, respectively. The CSR improved as much as 57% when using 1,000 iterations. From Figure 6, we can observe the improvements of DIR using 100 iterations over MBT in terms of dosimetric coverage. The 10 cases that did not directly meet our definition of clinical success had a *V*_95%_ for the prostate between 90% and 97% with a mean of 95% for DIR using 100 iterations, while for DIR using 500 iterations this range was between 93% and 97% with a mean of 96%. More details are given in the Discussion. See Figure 1 for an example.

**Table 5:** Percentage of elastix registrations that meet the dose constraints for the different contours.

	*****V***_**95**%_ ≥ 98%**				*****V***_**107**%_ ≤ 2%**		
	**Prostate**	**Seminal vesicles**	**Lymph nodes**	**CSR**	**Prostate**	**Seminal vesicles**	**Lymph nodes**
MBT	81.5	62.0	90.2	50.0	100.0	100.0	100.0
100	89.2	78.5	97.9	68.8	100.0	100.0	100.0
500	89.2	88.2	97.9	77.4	100.0	100.0	100.0
1,000	89.2	88.2	98.9	78.5	100.0	100.0	100.0
2,000	90.3	88.2	97.9	77.4	100.0	100.0	100.0

*Conservative success rate (CSR) refers to the percentage of registrations for which all target volumes (the prostate, seminal vesicles and lymph nodes) meet the dose constraints*.

## 4. Discussion

The purpose of this study was to investigate if automatic recontouring for prostate cancer IMPT would be possible, considering the clinical requirements for accuracy, robustness and speed. The overall goal of online adaptive IMPT is to be able to treat with a small margin (in this study 2 mm for the prostate and 3.5 mm for the seminal vesicles and lymph nodes) to spare OARs. This can only be done by daily re-planning, otherwise coverage loss or underdosage may occur, which is unacceptable. Such daily re-planning warrants automatic recontouring, in this study by DIR. To quantify the dosimetric impact of such re-planning, we not only performed a geometric validation but moreover a dosimetric validation, to assess the imperfections of automatic recontouring in terms of the clinically more relevant dose coverage. The chosen endpoint of our validation is therefore *V*_95%_ ≥ 98% for each of the target volumes. In general, the registration package elastix can automatically re-contour repeat CT scans of the prostate with a desirable accuracy in 3 s in 68.8% of cases. This compares favorably with MBT, which only obtained a success rate of 50%. elastix outperformed ANTs in this use case, in terms of registration accuracy as well as runtime efficiency. The parameter settings of ANTs were taken from Cao et al. (44), and then tuned with some efforts on a subset of the data. It may be that settings can be found that yield better registration accuracy, as the tuning was not exhaustive. Due to the nature of the ANTs algorithm, runtime performance however is highly in favor of elastix, an important aspect in online adaptive IMPT.

Several aspects were important for registration performance: (1) A correct initialization of the registration was found to be important to obtain desired accuracies. Alignment of bony anatomy, as used in this study, yielded satisfactory results (1, 52), but exploitation of the implanted gold markers could also be an option (50); (2) The couch is disturbing the registration and should therefore be removed by masking or cropping. In this study both were used, where cropping was also beneficial for runtime performance; (3) We observed performance differences when increasing the number of iterations, however, the effects are quite small, a few tenth of a millimeter. For example, the DSC of the lymph nodes decreased somewhat with increasing the number of iterations while the DSC of other organs increased, which may be caused by the deformation of the bladder which affected the lymph node region as well, see Table 1. (4) As we had compared the registration accuracy with and without mask, we found that masking is helpful for small volume organs such as the seminal vesicles and rectum, while no differences were observed for the prostate and lymph nodes. This finding is consistent with previous studies (1, 10).

In this study special attention was given to the registration runtime in relation to achieved accuracy, determined by the number of iterations used by DIR. Overall, registration accuracy increased only slightly when gradually increasing the number of iterations from 100 to 2000, suggesting that convergence was in most cases obtained already at 100 iterations. Only for the seminal vesicles an improvement in dose coverage was observed when using 500 iterations, see Table 5. The geometrical success rate as expressed by the percentage of registrations with an MSD below the slice thickness of 2 mm was 96% for the prostate. Clinical success rate, expressed by the dose coverage criteria, was 89% for the prostate. This means that in a high percentage of cases the automatically generated contours can be directly used for online adaptive IMPT, without the need for more time-consuming interaction. For those patients, smaller margins can be used and less robustness can be included than when using conventional non-adaptive planning [typically 7 mm margins (3, 49, 50)], resulting in less dose for the OARs and potentially less complications for the patients. For the remaining cases interaction is warranted, for example by manually supplying corresponding points at anatomical regions that require improvement (53, 54).

From the 93 registrations that were assessed in terms of target coverage, 10 did not directly meet the dose conformity constraints for the prostate. These cases were inspected visually, and we found that 2 cases had many gas pockets in the rectum, while for the other 8 cases no apparent reason was found. Note that no rectal balloon or SpaceOAR hydrogel was used for the current study. For cases with many gas pockets we may consider specialized DIR methodology using an intensity modification technique (55). The MSD of these two cases was around 2.3 mm, and therefore also did not meet the geometrical criteria. Of the 8 remaining cases, one case had a *V*_95%_ of 97.99%, which increased to >98% when 500 or more iterations were used. Two cases had a *V*_95%_ around 97% for all settings, which is very close the threshold of 98%; both had an MSD of 1.3 mm, meeting the geometrical criterion for success. Two cases had a *V*_95%_ of 96%, which improved to 98% and 99% when 500 iterations or more were used. The remaining three cases obtained a *V*_95%_ in the range 92–96% for the prostate and an MSD in the range 1.6-1.8 mm, so were not far from success.

In order to use the open source package elastix in a clinical setting, several aspects should be considered. Although the runtime of DIR is reduced to seconds in the current study, other steps in the entire replanning process should also be considered to shorten the procedure and improve the quality. Special attention should be paid to the intrafraction motion of the CTV between the time of CT image acquisition and the planned dose treatment delivery (56, 57). For online adaptive IMPT, one could use the daily imaging techniques, such as CT and MR, to obtain the new position of hard or soft tissues. They can also use the implanted fiducial markers, rectal balloon or SpaceOAR hydrogel to guide the new treatment delivery (22, 58–64). All of these require a reasonable margin to tackle the randomness of the intrafraction motion of the CTV. With the help of reduced registration time, even smaller margins (less than 2 or 1 mm) could be possible (65) and other uncertainties are under control. Quality control is another important issue, which can be performed via visual inspection of the generated contours, but assistance by automatic techniques for uncertainty estimation of image registration may be of interest (66, 67). These techniques may pinpoint areas of possible misregistration, thus enabling a quicker assessment of registration quality. Contours generated by elastix could be directly used in 89% of cases, meaning that for 11% of cases manual assistance or fall-back strategies are needed. Registration can for example be efficiently improved by manually indicating a few landmarks on segmentation boundaries (54). Robustness may be further improved by taking into account automatic estimates of the bladder (68) in the registration by optimizing a joint functional. In this study we used clinical quality repeat CT scans, which assumes the availability of an in-room CT-on-rails system (69–71). Since such a system is not available in all hospitals, alternatively Cone Beam CT (CBCT) may be used in-room (20, 72). However, the reduced soft-tissue contrast of CBCT images may increase the uncertainty of DIR, which therefore may influence the quality of the IMPT plans. Lastly, the registration time assessed in this paper is determined by the number of iterations, which is not case-specific (36, 73). For cases that are geometrically close, DIR may finish the task with less than 100 iterations, while for difficult cases the number of iterations may be much larger. An adaptive stopping condition for stochastic gradient descent, such as considering a moving average of the noisy cost function values (or gradient), may remedy this. Moreover, a further reduction in runtime may be obtained with the help of a graphics processing unit (GPU) and other computational techniques (11, 74–76).

## 5. Conclusion

In this study we showed that the open source registration package elastix can automatically re-contour repeat CT scans of the prostate in 3 s, yielding treatment plans that directly meet the dose conformity constraints in 78.5–97.9% of cases, and a geometrical criteria of success in 96% of cases. This software may therefore facilitate online adaptive proton therapy of prostate cancer, enabling a reduction in treatment margins and robustness that needs to be included in the treatment plan.

## Data Availability Statement

The CT-data with contours were collected at Haukeland University Hospital, Bergen, Norway and were provided to us by responsible oncologist Svein Inge Helle and physicist Liv Bolstad Hysing. Requests to access this dataset should be directed to Liv Bolstad Hysing, liv.bolstad.hysing@helse-bergen.no.

## Ethics Statement

The trial had been approved by the local ethical committee of REC West, the western Norway regional committee for medical and health research ethics (number 2006-15727). Written informed consent was obtained for participation in the study.

## Author Contributions

YQ contributed to experimental design, implementation, geometric evaluation, interpretation of the data, and the writing of the manuscript. TJ contributed to the implementation, experiments on dosimetric evaluation, and interpretation of the data. MH, BL, and MS were involved in experimental design, in the writing of the manuscript, and have read and approved the final version.

### Conflict of Interest

TJ reports grants from Varian Medical Systems, Pao Alto, US, during the conduct of the study; grants from Elekta AB, Stockholm, Sweden, grants from Accuray, Sunnyvale, US, outside the submitted work. MH reports grants from Varian Medical Systems, Pao Alto, US, during the conduct of the study; grants from Elekta AB, Stockholm, Sweden, grants from Accuray, Sunnyvale, US, outside the submitted work. The remaining authors declare that the research was conducted in the absence of any commercial or financial relationships that could be construed as a potential conflict of interest.
